# Supplementary material for: Flat bands, non-trivial band topology and rotation symmetry breaking in layered kagome-lattice RbTi3Bi5
Source: Nat Commun. 2023 Aug 14;14:4892. doi: 10.1038/s41467-023-40515-3 (PMC10425367; doi:10.1038/s41467-023-40515-3)
Supplement: Supplementary file 1 — Supplementary Information [file 41467_2023_40515_MOESM1_ESM.pdf]

Supplementary Information for
"Flat bands, non-trivial band topology and rotation symmetry breaking in layered kagome-lattice $RbTi_3Bi_5$"

Zhicheng Jiang[1 †], Zhengtai Liu[1,2 † *], Haiyang Ma[3,4 †], Wei Xia[3,4 †], Zhonghao Liu[1], Jishan Liu[1,2], Soohyun Cho[1], Yichen Yang[1], Jianyang Ding[1], Jiayu Liu[1], Zhe Huang[3], Yuxi Qiao[1], Jiajia Shen[1], Wenchuan Jing[1], Xiangqi Liu[4,5], Jianpeng Liu[3,4 *], Yanfeng Guo[3,4 *], Dawei Shen[1,5 *]

[1]*State Key Laboratory of Functional Materials for Informatics, Shanghai Institute of Microsystem and Information Technology, Chinese Academy of Sciences, 200050 , Shanghai, China*

[2]*Shanghai Synchrotron Radiation Facility, Shanghai Advanced Research Institute, Chinese Academy of Sciences, Shanghai 201210, China*

[3]*School of Physical Science and Technology, ShanghaiTech University, 201210, Shanghai, China*

[4]*ShanghaiTech Laboratory for Topological Physics, ShanghaiTech University, 201210, Shanghai, China*

[5]*National Synchrotron Radiation Laboratory, University of Science and Technology of China, 42 South Hezuohua Road, Hefei, Anhui 230029, China*

∗ *Corresponding authors: Dawei Shen (dwshen@ustc.edu.cn); Yanfeng Guo (guoyf@shanghaitech.edu.cn); Jianpeng Liu(liujp@shanghaitech.edu.cn); Zhengtai Liu (ztliu@mail.sim.ac.cn)*

† *These authors contributed equally to this work.*

Supplementary Notes 1-6

Supplementary Note 1. Characterization of single crystal. $RbTi_3Bi_5$ single crystals have a typical size of 3 $\times$ 5 $\times$ 0.5 mm^3 [Fig. S1(a)], all the sample needs to prepare in an argon-filled glove box to prevent the oxidation and hydrolysis of Rb. Single-crystal XRD was collected using a Bruker D8 x-ray diffractometer with Cu K_α radiation (λ= 0.15418 nm) at room temperature. The XRD pattern of a $RbTi_3Bi_5$ single crystal [Supplementary Figure 1(b)] reveals that the crystal surface is parallel to the (00l)-plane. The estimated c-axial lattice constant is about 9.065 Å, close to the previously reported values. We conducted core-level spectroscopy with the photon energy of 120 eV. In Supplementary Fig. 1(c), we present the core-level photoemission intensity plot of $RbTi_3Bi_5$ where the characteristic peaks of Rb-4s/4p, Ti-3s/3p, and Bi-5d orbitals are clearly observed, with no extra peaks observed, suggesting the phase pure $RbTi_3Bi_5$ single crystals.

Supplementary Note 2. Overview of band structures. We performed the measurements of the constant energy contours from Fermi Level (E_F) to E_F-1.0 eV with 66 eV photon, as shown in Supplementary Fig. 2. It shows typical band structure of kagome patterns, which consists of a circle-like electron pocket (α band), a hexagonal-like electron pocket (β band) around Γ point, another larger hexagonal-like electron pocket (γ band) encircle β band and with 30° respect to it is also been observed, this γ band then cross Fermi surface again around K point, and constitutes a triangle-like electron pocket around K point. In contrast, there is a rhombic hole pocket (δ bands) around M points. The orbital character of the highlighted bands is presented in the Supplementary Fig. 3. The Fermi surface are mainly constituted by d-orbits of Ti-atoms and p-orbits of Bi atom, the calculated orbital distribution at Fermi surface is presented in Supplementary Fig. 3(b), and more detailed orbital calculations are presented in Supplementary Fig. 3(d)-(e). According to the calculation, we can summarize the orbital characters of different bands in Supplementary Fig. 3(c), where the inner circular pocket (red) around Γ point mainly originates from the Bi p_z orbital, the hexagon-like one (green) is from Ti d_{xz}/d_{yz} orbitals, the another hexagon-like pocket (yellow) is mainly from Ti $d_{xy}/d_{x^2-y^2}$ orbitals, and the rhombic-like and triangle-like bands (gray) are mainly attributed to Ti p_x/p_y orbitals.

Supplementary Note 3. Features of flat bands. We inspected the intensity plots and corresponding second derivative plots of $RbTi_3Bi_5$, $CsTi_3Bi_5$, and KTi_3Bi_5 with 66, 86, and 74 eV photons, respectively, along high symmetry-paths, which correspond to M-K-Γ-K-M lines, as shown in Supplementary Figure 4. This flat band seemingly spans the entire BZ and is distinct from our calculation, in which the flat band is absent in the regions along $\Gamma - K$ and $\Gamma - M$, as shown in Figure 1(e-f). Note that we have identified such similar flat band feature in all ATi_3Bi_5, as shown in Supplementary Figure 4. Meanwhile, we note that the position of full-k-space flat bands has a slight shift with the change of the A site elements. It's worth to notice that both the energy position of flat bands shift with the flat band at around $K - M$. Such a feature is especially conspicuous in KTi_3Bi_5, in which two prominent shadow flat bands (-300/-580 meV) coincide with the flat bands around $K-M$ at the same binding energies [Supplementary Figure 4(c) and (f)]. As shown in Supplementary Figure 5(b) and (c), the constant energy surface taken at E_F -0.3 eV and E_F -0.58 eV of KTi_3Bi_5 both show high density of states around the edge of Brillouin zones. These flat bands can also be clearly observed along the high symmetric $K - M - \Gamma - K - M$ direction; we marked these two flat bands with red arrows in Supplementary Figure 5(e). Besides, we note that when we alter the photon energy to the $L - H - A - H - L$ direction, the shadow flat band at E_F -0.58 eV becomes much clearer. Additionally, both flat bands extend a feature with low intensity to the Γ point, which is not visible in our calculations. The shadow flat band at around E_F -0.3 eV can be visible in the raw spectrum [Supplementary Figure 5(d)] and second-derivation spectrum [Supplementary Figure 5(e)], respectively. Given that the binding energy of this low intensity flat bands

always coincides with that of DFT predicted small flat bands around K and M, namely -0.25 eV in $CsTi_3Bi_5$ and $RbTi_3Bi_5$ and -0.30/-0.58 eV in KTi_3Bi_5, the shadow flat band across the entire Brillouin zone should tightly linked with the flat bands confined in $K-M$ direction.

The origin of such a shadow flat band in ATi_3Bi_5 should be closely linked with the small flat band. In this way, we conjecture that there might exist several possible origins of this shadow flat band. Firstly, we speculate that it might originate from some rather localized states in ATi_3Bi_5, e.g., parts of localized titanium $3d$ electrons due to spin frustration or impurity states with relatively lower intensity and dispersionless feature. Secondly, this feature might be related to the k_z broadening of electronic states at Γ. Although the in-plane electron hopping is confined in kagome lattices, the interlayer electron hopping is still significant in real three-dimensional stacked kagome lattices, resulting in dispersive bands along the k_z direction. Our DFT calculations have indicated that the band structure around Γ is largely dispersive along k_z, which would result in a band structure evolution from the electron-like to "drumhead"-like, as shown by the highlighted area around Γ/A in Supplementary Figure 6. Since our ARPES measurements were performed mainly using VUV photons, the mean escape lengths of resulting photoelectrons are limited. Consequently, the photoemission spectra may be more influenced by the k_z broadening effect. However, we note that the origin of this shadow flat band needs more research to pin down.

Supplementary Note 4. Temperature dependent measurements. We performed temperature-dependent ARPES experiments on $RbTi_3Bi_5$, and our results further verified the absence of charge density waves. By comparing the band dispersions taken along high-symmetry direction at low temperature (11 K) and high temperatures (200 K), as shown in Supplementary Figure 9, we find all the band features does not show any changes.

Supplementary Note 5. Autocorrelation of ARPES spectra (AC-ARPES). The original data are extracted from constant energies counters extracted from different binding energies integrated with the spectral weight over E_F $\pm$ 25 meV. The AC-ARPES in Fig. 5c-e and Supplementary Fig. 11-14 are calculated from the ARPES intensity maps with following equation

$$C(\mathbf{q}) = \alpha \int^{BZ} A(\mathbf{k}, E)\, A(\mathbf{k}+\mathbf{q}, E)\, d\mathbf{k} \qquad \text{(Supplementary Equation 1)}$$

where $A(\mathbf{k}, E)$ is the spectral function at binding energy E at $\mathbf{k}$ points in the BZ, and α is constant [1–4]. In Supplementary Figure 11, we compared the AC-ARPES spectra extracted from E_F of $RbTi_3Bi_5$ and KV_3Sb_5, both of them shows anisotropic intensity distribution. We have also compared the AC-ARPES extracted from E_F to E_F-0.5 eV by step of 0.1 eV, as shown in Supplementary Fig. 13. It shows hint of anisotropic intensity distribution near E_F, but on higher binding energies, the six-fold symmetry recovered.

In Supplementary Fig. 12a, we identified the scattering vectors according to the ARPES Fermi surface of $RbTi_3Bi_5$ ($\mathbf{q}_1$ $\mathbf{q}_4$), and we append them on the autocorrealtion map, as shown in Supplementary Fig. 12b. As the band structures on Fermi surface are complex, to distinguish the different scattering routes, we further extract the main bands from the models and redo the autocorrelation simulation, as shown in Supplementary Fig. 12c to 12f. The scattering vectors ($\mathbf{q}_1$, $\mathbf{q}_1^{'}$, $\mathbf{q}_1^{''}$) origin from the larger hexagon pocket around Γ point primarily give rise to a bright line surrounding the Γ point, as shown in Supplementary Fig. 12c; the scattering vectors ($\mathbf{q}_2$, $\mathbf{q}_2^{'}$) origin from the smaller hexagon pocket around Γ point primarily give rise to the bright spots around K point, as shown in Supplementary Fig. 12d; the scattering vectors ($\mathbf{q}_3$) between α band around Γ point and δ bands around M point gives rise to the bright spots near M points, as shown in Supplementary Fig. 12e; the scattering vectors ($\mathbf{q}_4$) between those triangle

and rhombic-like pockets on the edges of Brillouin zone mainly contribute to the three parallel lines along $\Gamma - M$ directions, as shown in Supplementary Fig. 12f.

Additionally, we have also demonstrated the AC-ARPES at 200 K in Supplementary Figure 14, which is similar to the data take at low temperature [Fig. 5c-d].

Meanwhile, we also attempted to measure rotated samples, as shown in Supplementary Figure 15. When the sample was rotated by -15° and -30°, both corresponding autocorrelation results rotate along with the sample. In particular, the characteristic feature of anisotropy in the amplitudes between the three nominally identical directions keeps rotating by the same degrees, as shown in Supplementary Figure 15. We extract the intensity distribution curves along three different $\Gamma - M$ directions in Supplementary Figure 15c. We observed a noticeable intensity peak at the position indicated by $\mathbf{q}_3$ in two out of the three directions (yellow and blue dashed cut lines). While the spectra weight on the three parallel lines appeared to be fixed during sample rotation, influenced by the polarization effect, the intensity peaks associated with $\mathbf{q}_3$ still rotated with the sample. These $\mathbf{q}_3$ peaks were distinguishable from the yellow and blue cut lines but indistinguishable from the red one in -15° and -30° rotated samples, as depicted in Supplementary Fig. 15(f) and 15(i). This result unambiguously indicates that the matrix element effects should not dominate the anisotropy in the autocorrelation. We speculate that this may be attributed to a small distortion of the δ-bands, which are primarily contributed by Bi $p_{x,y}$ orbitals and hybrid orbitals of Bi $p_{x,y}$ and Ti d_{xy} orbitals. These bands could undergo deformation in nematic phases. However, due to the complexity of Fermi surface scattering and the small magnitude of the distortion, we were unable to fully pin down the exact origin of the rotation symmetry breaking, which still wait for further exploration.

To validate the feasibility of autocorrealtion computational approach in "135" series kagome materials, we conducted a similar investigation on KV_3Sb_5 [5, 6], a known kagome metal with nematic phase. In Supplementary Fig. 16(a), we have marked the different scattering channels by double-head arrows along different $\Gamma - M$ directions: $\mathbf{q}_1$ represents the scattering from the circular-like band around Γ to the folded circular-like band around M, $\mathbf{q}_2$ represents that from the circular-like band around Γ to the "bow"-like band around M. The autocorrelation calculation enables us to transfer the k-space spectrum into q-space, as demonstrated in Supplementary Fig. 16(b). Since the C_6 rotation symmetry is broken in KV_3Sb_5, the band structure of KV_3Sb_5 also exhibits an anisotropic feature. To highlight the anisotropic feature in the q-space image of autocorrelation, we directly compared the three intensity curves taken along Cut-1, Cut-2 and Cut-3, as shown in Supplementary Fig. 16(c). We observed that the intensity at $\mathbf{q}_2$ along all three directions are approximately equal, while the adjacent $\mathbf{q}_1$ peak along Cut-3 is much higher than those along Cut-1 and Cut-2. Moreover, the anisotropy feature can be more clearly seen from the autocorrelation result of the constant energy intensity map at E_F - 0.2 eV, as illustrated in Supplementary Fig. 15(d)-(f). We note that the intensity of $\mathbf{q}_2$ peaks is nearly equal for all three directions. However, the $\mathbf{q}_1$ peak is rather outstanding along Cut-3 while it is evidently suppressed along both Cut-1 and Cut-2. Both bulges at $\mathbf{q}_1$ taken along Cut-3 for E_F and E_F - 0.2 eV reflect relatively stronger correlation between the pristine and CDW folded bands along $\mathbf{q}_1$ for KV_3Sb_5, unambiguously indicative of the breaking of original C_6 symmetry therein.

Supplementary Note 6. Calculation of topological index. The $\mathbb{Z}_2$ topological invariant is computed via the parity products at the time reversal invariant momentum (TRIM) points. Although the inversion symmetry compels the 122 and 124 bands to cross along $\Gamma - A$, thereby inhibiting the formation of a continuous gap between these two bands, this criterion can still be applied here according to the theory of symmetry indicators [7, 8]. According to this theory, parity eigenvalues at the TRIM points can be used to identify band topology, irrespective of the presence or absence of a continuous band gap. For ATi_3Bi_5, which exhibits both time-reversal and space-inversion symmetries,

the following general form expresses its topological invariant:

$$\chi_{BS} = \mathbb{Z}_2^3 \times \mathbb{Z}_4 \quad \text{(Supplementary Equation 2)}$$

where the three $\mathbb{Z}_2$ indices correspond to the three weak indices of the Fu–Kane criterion and the strong $\mathbb{Z}_2$ index is elevated to a $\mathbb{Z}_4$ factor. One can still utilize the $\mathbb{Z}_2$ index to determine whether the compound is topological or not. However, to discern whether the compound is an insulator or semimetal, we concur that further analysis of the band crossings is necessary.

Supplementary Figures 1-11

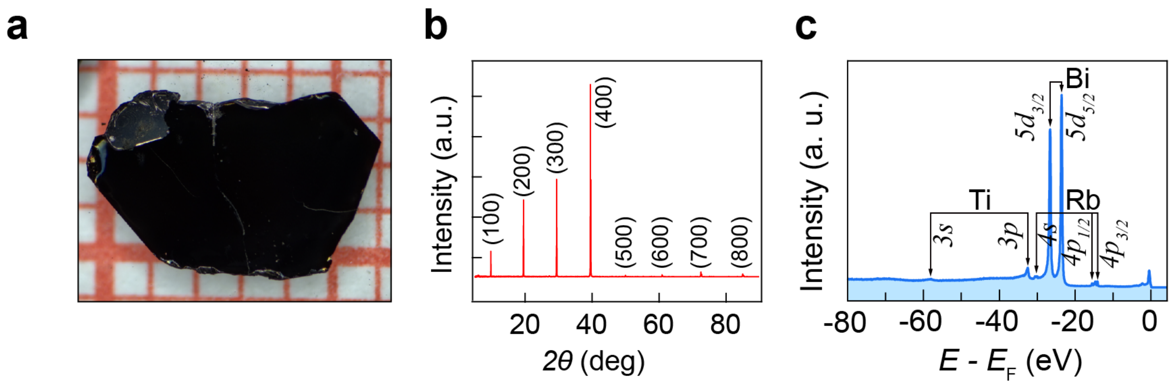

Supplementary Figure 1. **a** An optical micrograph of ab plane of $RbTi_3Bi_5$ single crystal on a 1mm grid; **b** Room temperature single crystal XRD patterns of $RbTi_3Bi_5$ and indexing; **c** ARPES core level energy distribution curve (EDC) of $RbTi_3Bi_5$.

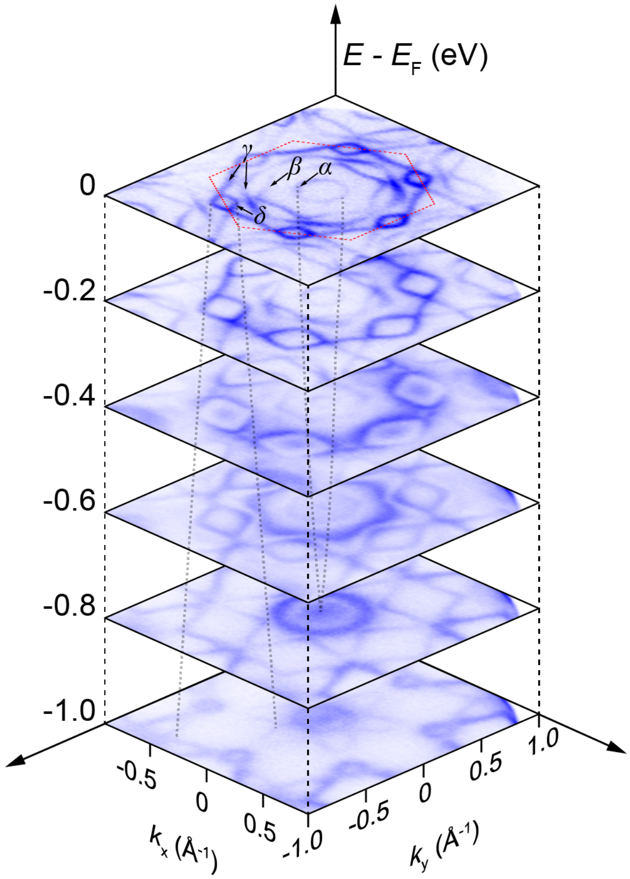

Supplementary Figure 2. Stacking plots of constant energy contours at different binding energies showing sophisticated band structure evolution as a function of energy.

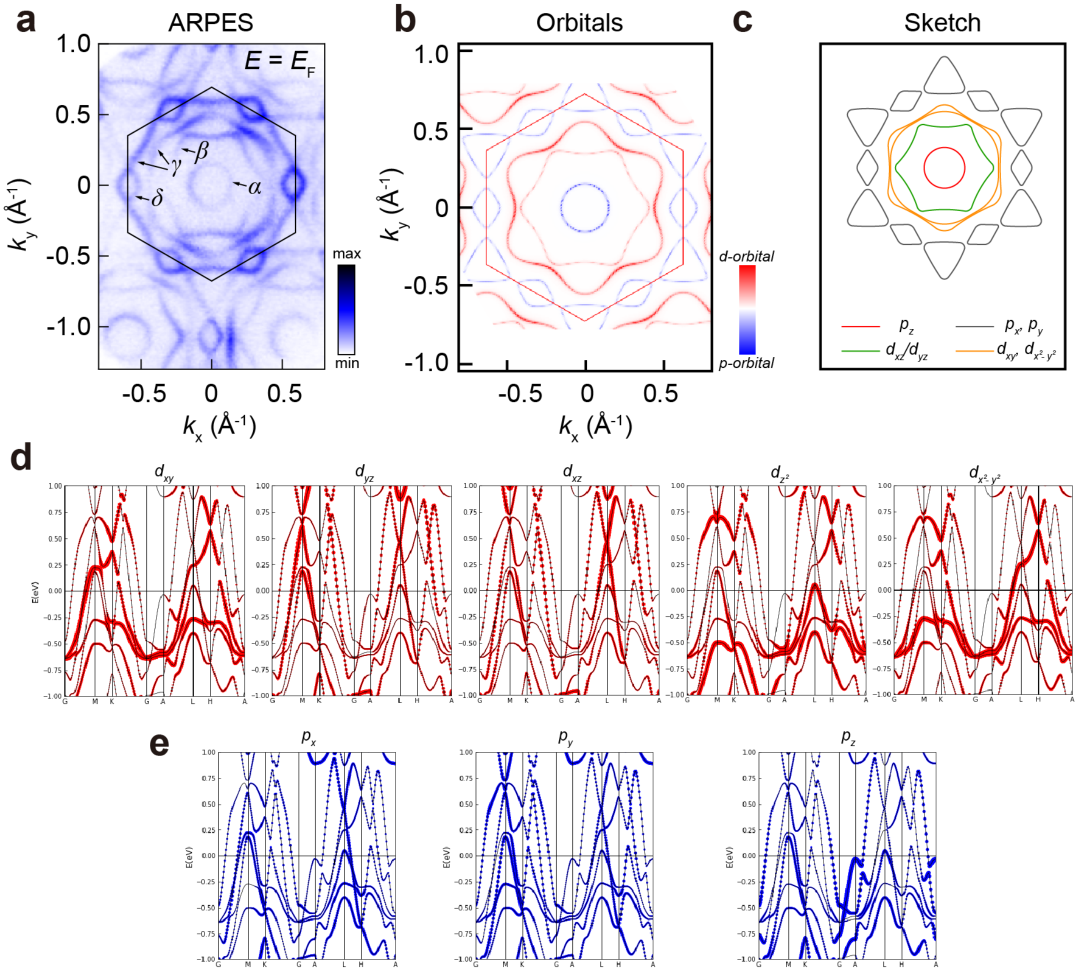

Supplementary Figure 3. **a** The ARPES map at Fermi surface of $RbTi_3Bi_5$; **b** The calculated distribution of orbitals on the Fermi surface of $RbTi_3Bi_5$; **c** The sketch plot of orbital characterization of $RbTi_3Bi_5$; **d** The calculated d-orbital distribution along high-symmetry cut line; **e** The calculated p-orbital distribution along high symmetry cut line.

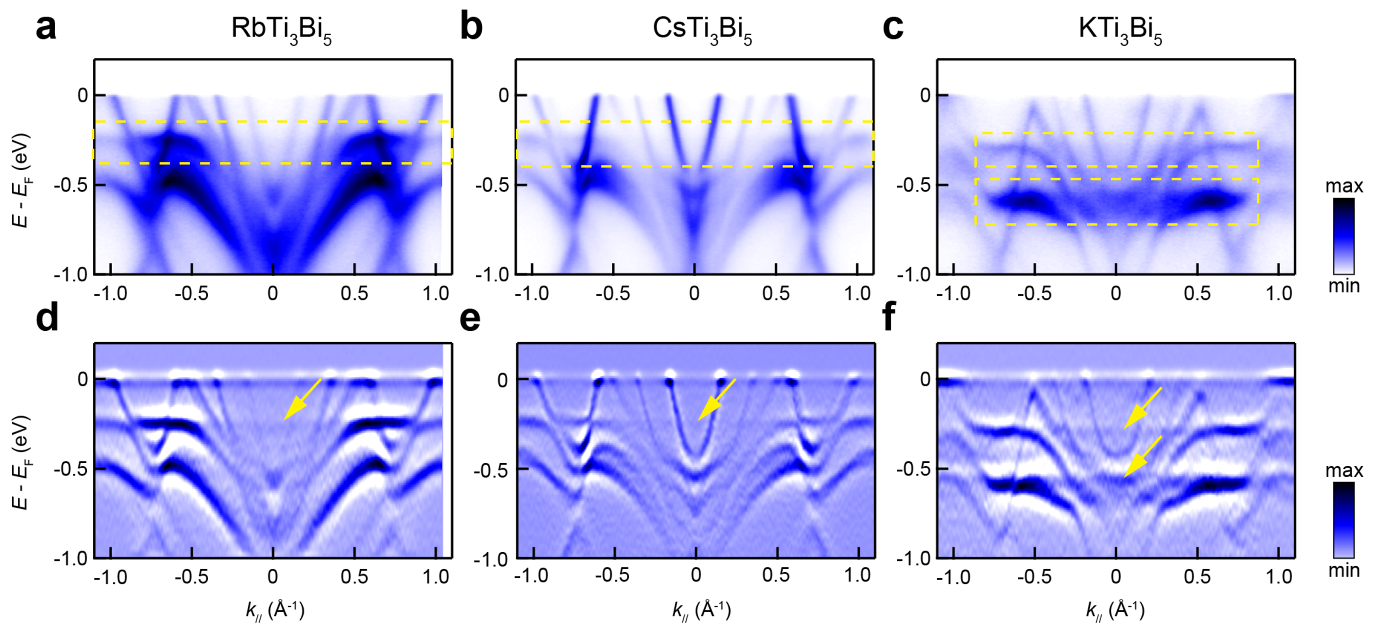

Supplementary Figure 4. **a-c** ARPES intensity plots of $RbTi_3Bi_5$, $CsTi_3Bi_5$ and KTi_3Bi_5 along M-K-Γ direction; **d-f** Corresponding second-derivation plots of **a-c**. The yellow dash frames denote the position of flat bands and arrows denote the shadow flat bands.

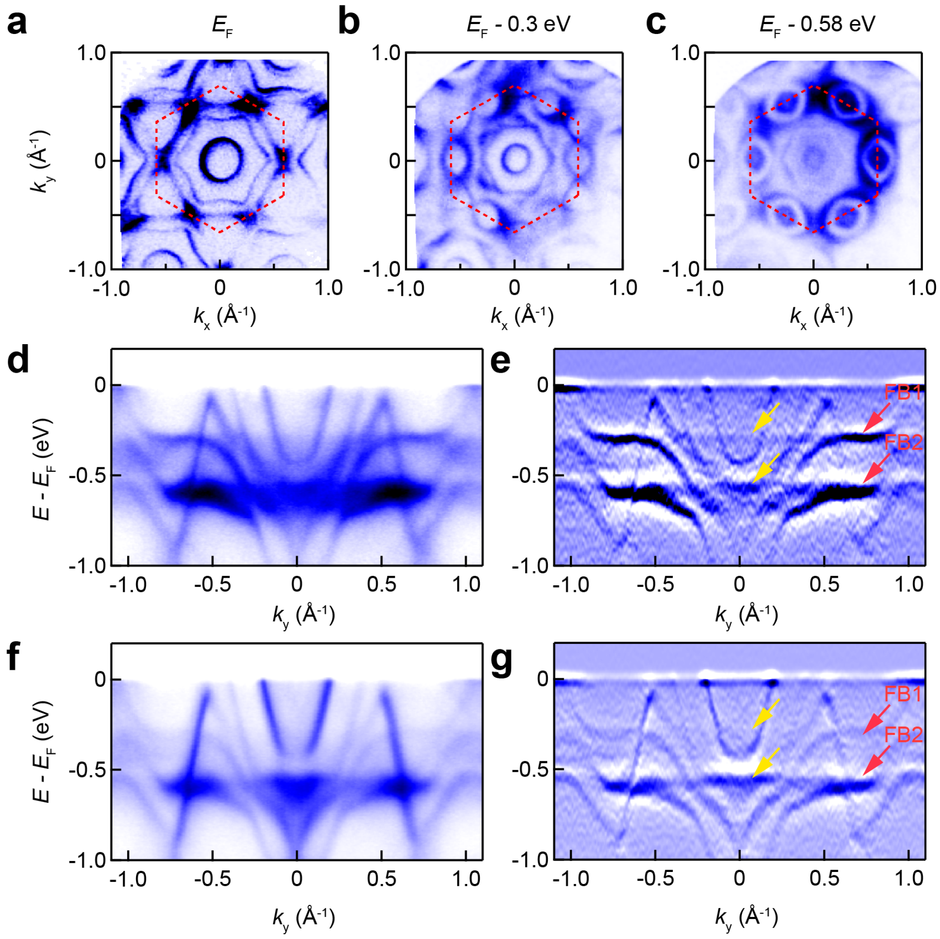

Supplementary Figure 5. **a-c** The constant energy maps taken at **a** E_F; **b** E_F -0.3 eV; **c** $E_F - 0.58$ eV of KTi_3Bi_5, respectively; **d** The band structure along $M - K - \Gamma - K - M$ measured at 74 eV; **e** The second-order derivation spectrum of **d**; **f** The band structure along $L - H - A - H - L$ measured at 86 eV; **g** The second-order derivation spectrum of **f**.

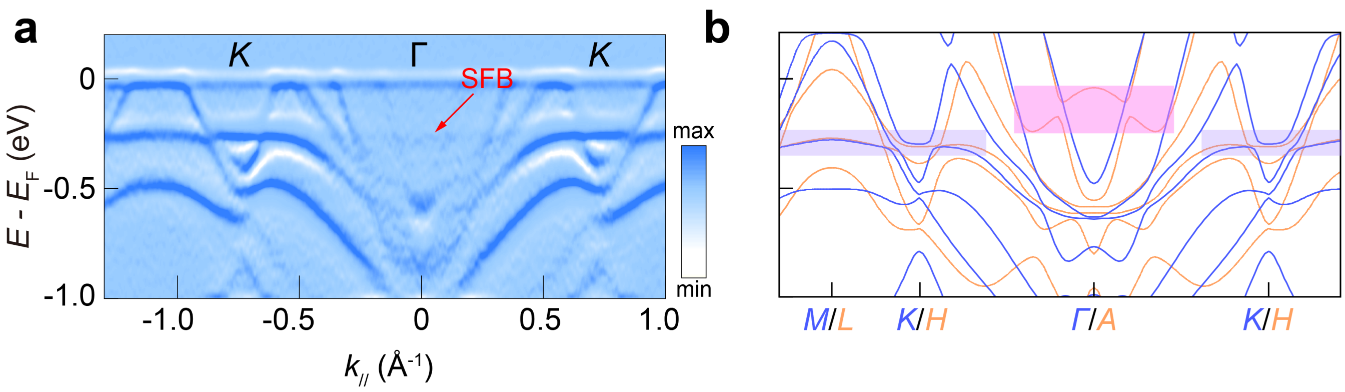

Supplementary Figure 6. **a** The second-order derivation ARPES spectrum cut along the $M - K - \Gamma - K - M$ direction; **b** The DFT calculated band structure of $RbTi_3Bi_5$, the blue lines calculated along the high-symmetry line $M - K - \Gamma - K - M$ direction and the yellow lines calculated along the high-symmetry line $L - H - A - H - L$.

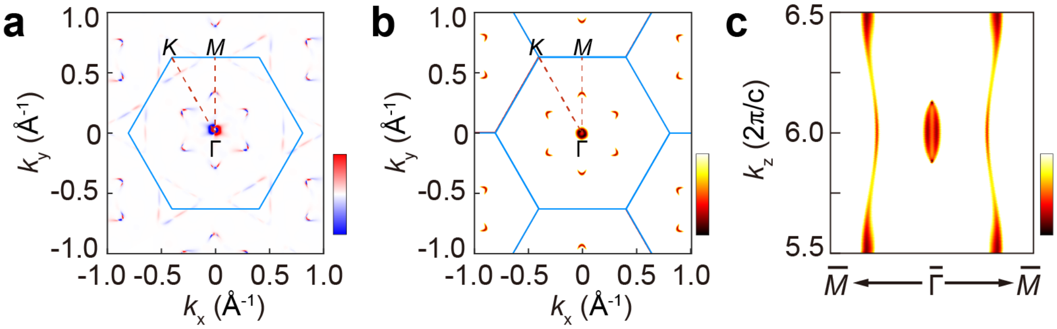

Supplementary Figure 7. **a** Calculated Berry curvature distributions on Γ-M-K plane at a binding energy of 0.25 eV; **b** Calculated in-plane distributions of type-II Dirac nodal point on Γ-M-K plane; **c** Distribution of Dirac nodal line on $k_{//}$-k_z plane along $\overline{\Gamma}$-$\overline{M}$ direction.

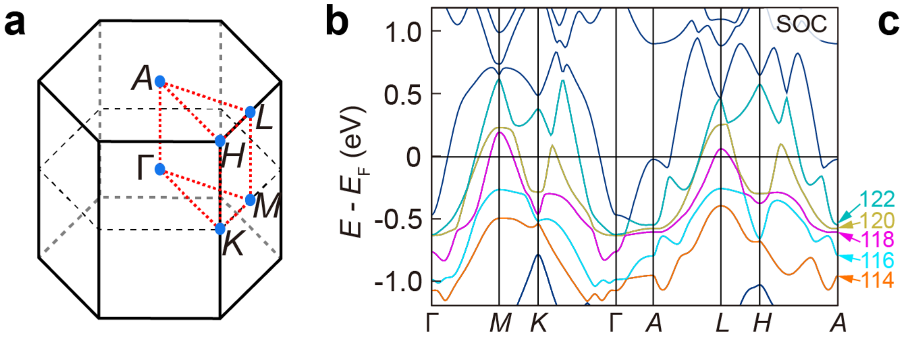

c

Band Index	Parity Eigenvalue				Z_2
	Γ	M	K	L	
114	+	-	-	+	0
116	+	-	+	+	1
118	-	-	-	+	1
120	+	+	-	-	0
122	+	-	-	+	0

Supplementary Figure 8. **a** Three-dimensional Brillouin zone of $RbTi_3Bi_5$ with high symmetry point (marked with blue dots and characters); **b** The calculated band structure of $RbTi_3Bi_5$ with spin-orbit coupling. Energy bands near Fermi level are drawn in different colors; **c** Product of parity of four time reversal invariant momenta and Z_2 indices of the bands near Fermi level. The colors of band indices are consistent with **b**.

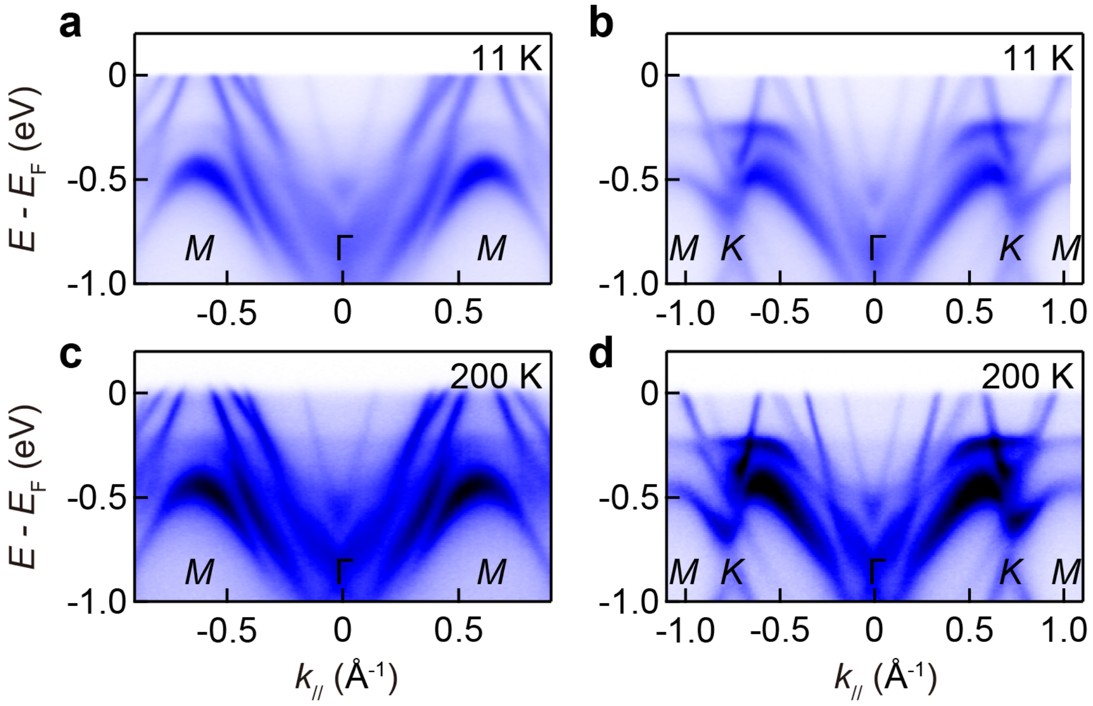

Supplementary Figure 9. ARPES intensity plots of $RbTi_3Bi_5$ taken along Γ-M and Γ-K-M. The data were collected at 11 K in **a, b** and 200 K in **c, d**, respectively.

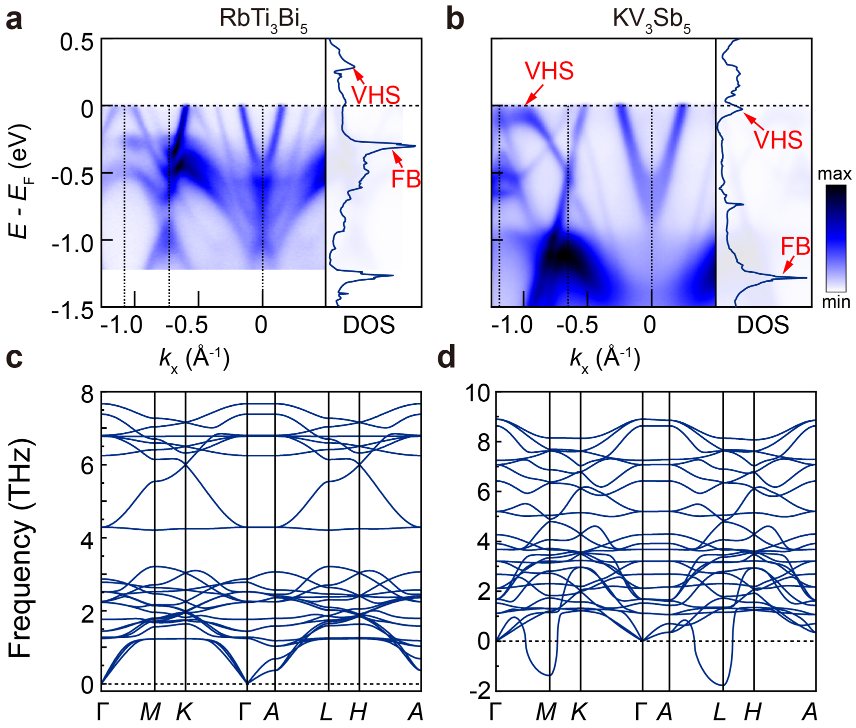

Supplementary Figure 10. **a, b** ARPES intensity plot of $RbTi_3Bi_5$ and KV_3Sb_5 along M-K-Γ. And the right panels show the corresponding total density-of-states (DOS) near the Fermi level. **c, d** Calculated phonon spectrum of $RbTi_3Bi_5$ and KV_3Sb_5 along a high-symmetry path.

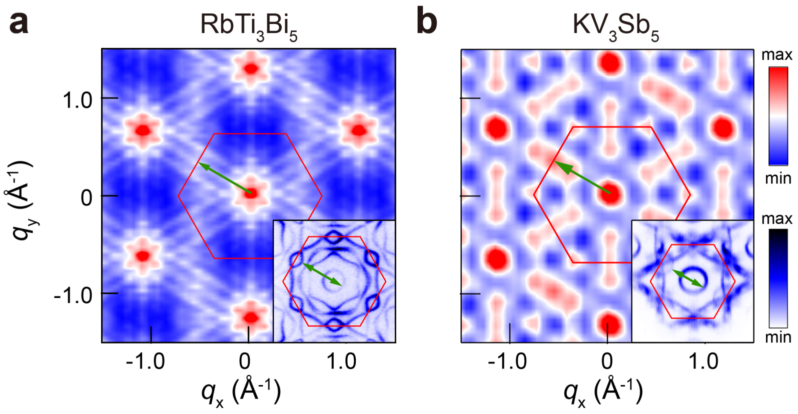

Supplementary Figure 11. **a, b** Autocorrelation maps obtained from the Fermi surface of $RbTi_3Bi_5$ and KV_3Sb_5. The insets are corresponding experimental Fermi surface maps with appended scattering channels q (green arrows).

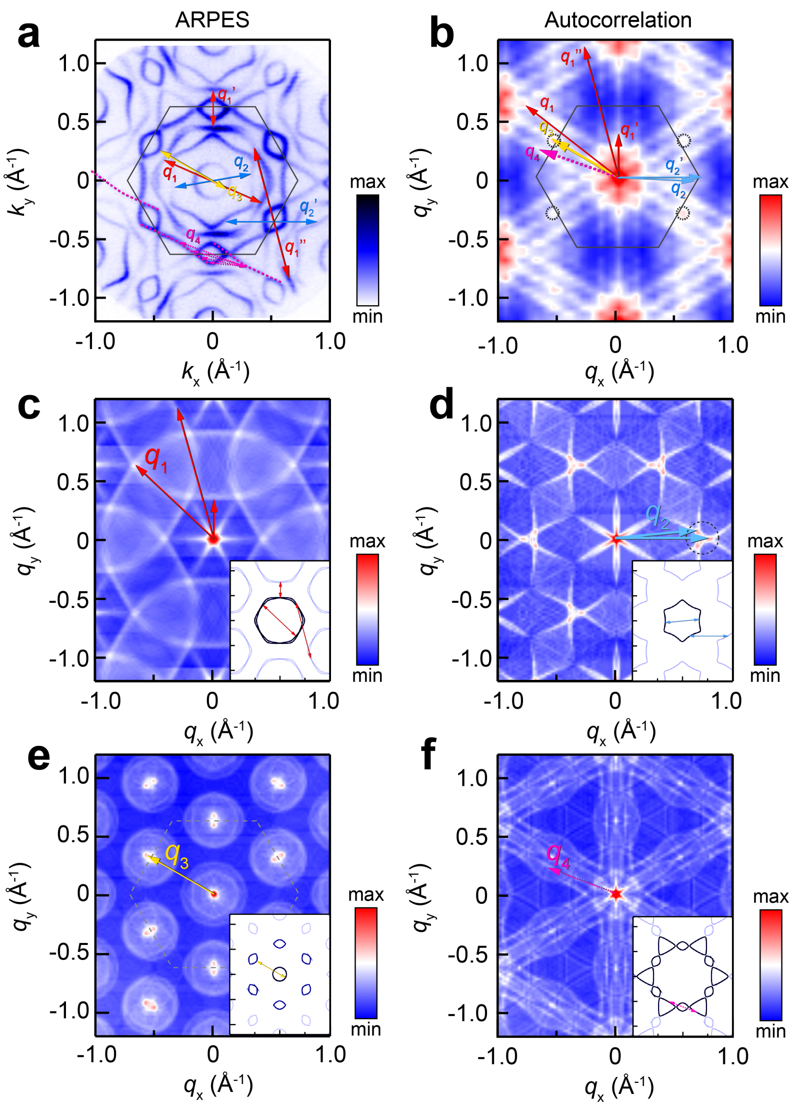

Supplementary Figure 12. **a** Fermi surface model extracted from the experiment Fermi surface map; **b** Autocorrelation calculation based on **a**; **c-f** Simulated autocorrelation result of extracted Fermi surface with band structure shown in the inset of **c-f**, respectively.

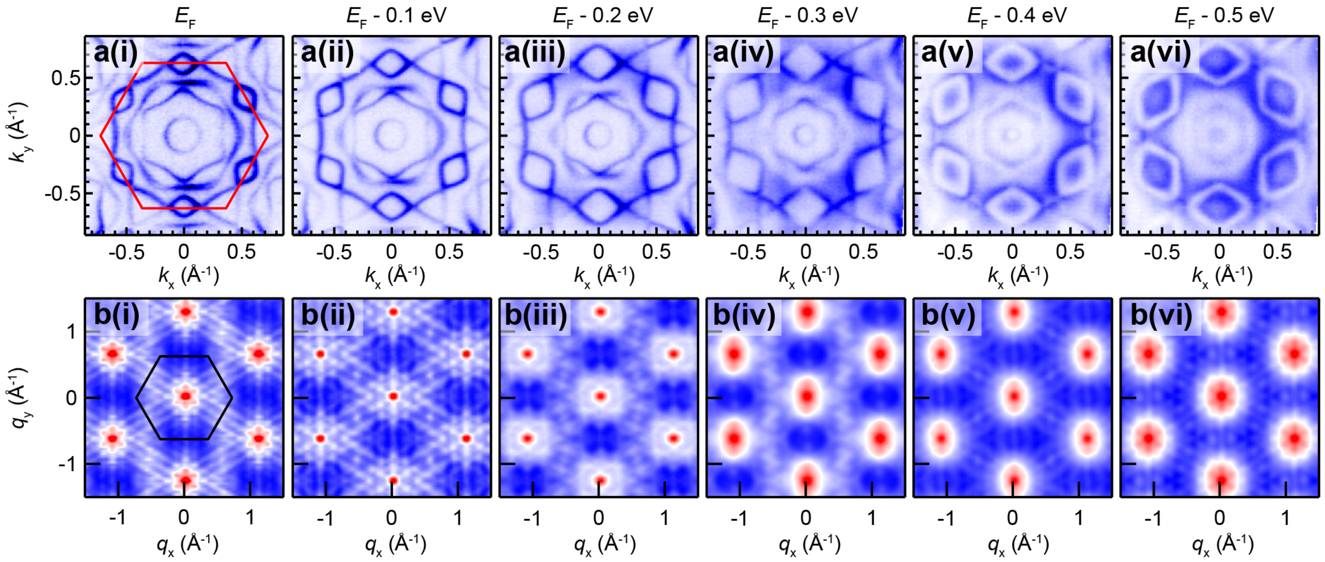

Supplementary Figure 13. **a(i)-a(vi)** ARPES constant energy contour maps and **b(i)-b(vi)** the corresponding autocorrelation plots of $RbTi_3Bi_5$ from E_F to E_F - 0.5 eV.

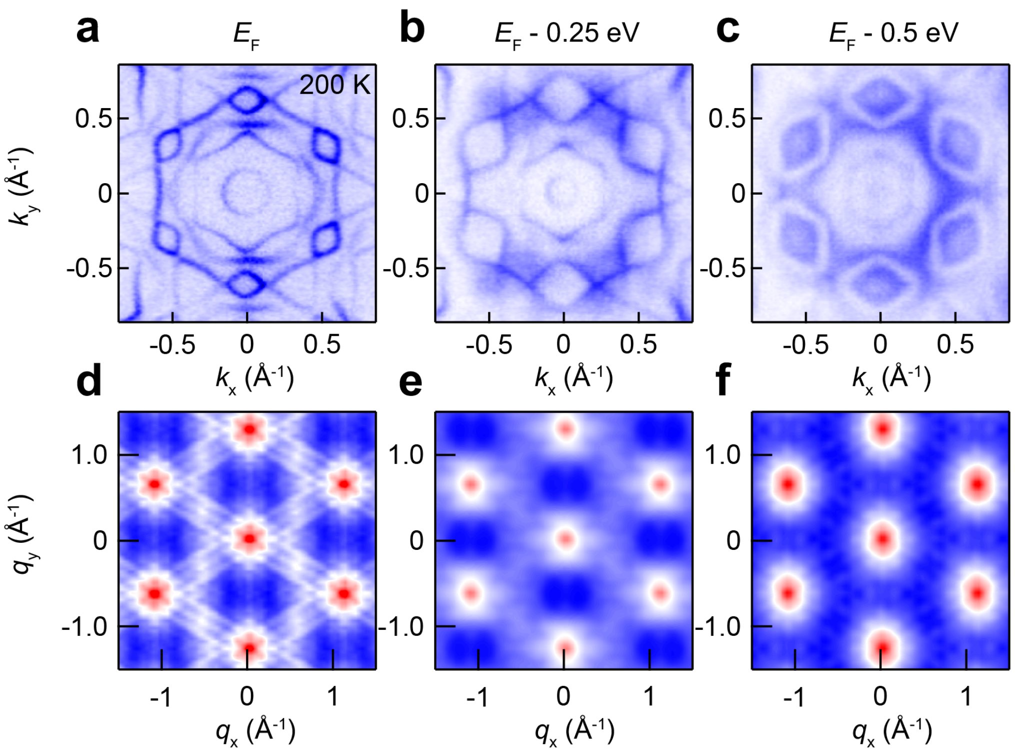

Supplementary Figure 14. **a-c** Experimental Fermi surface maps $RbTi_3Bi_5$ taken at E_F, E_F - 0.25 eV, E_F - 0.5 eV and 200 K. **d-f** Two-dimensional joint DOS results extracted from Fermi surface maps in **a-c**.

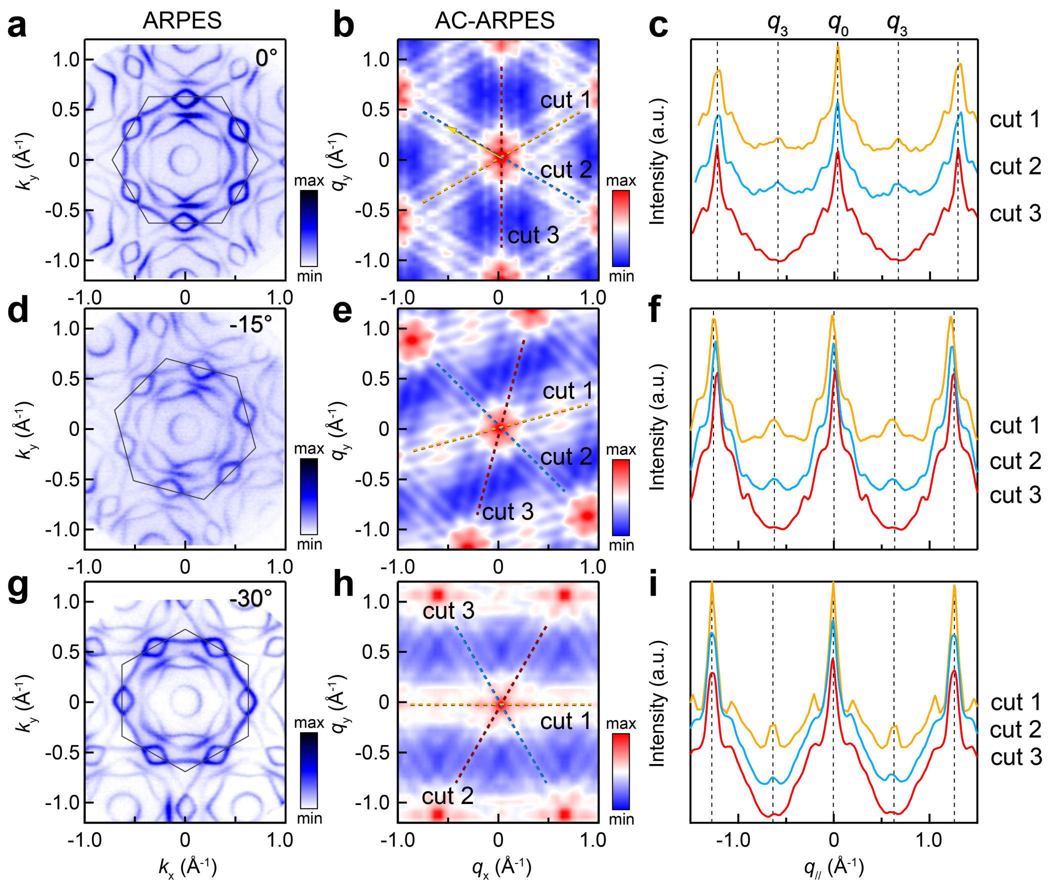

Supplementary Figure 15. **a, d, g** ARPES intensity maps taken at Fermi surface with (**a**) 0°, (**d**) -15° and (**g**) -30° respect to the analyze slit direction; **b, e, h** AC-ARPES spectra calculated from **a**, **d**, and **g** respectively; **c, f, i** The intensity distribution curves along cut 1-3 in **b**, **e** and **h**.

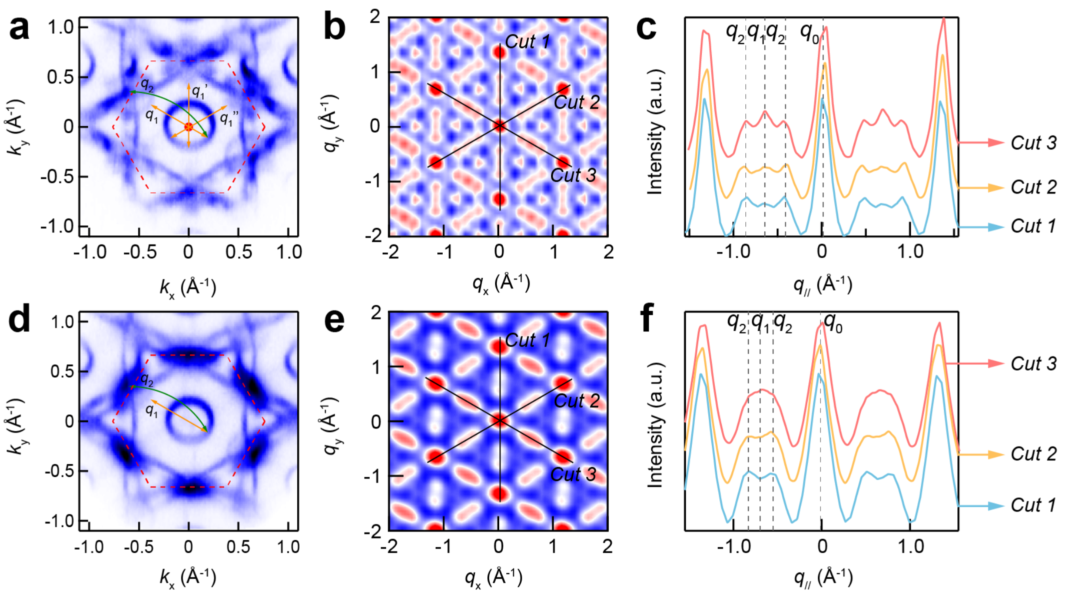

Supplementary Figure 16. **a** The ARPES map of KV_3Sb_5 at Fermi surface and 14 K; **b** The autocorrelation results calculated from **a**; **c** The intensity distribution curves along cut 1, cut 2 and cut 3 in **b**; **d** The ARPES map of KV_3Sb_5 at $E_F - 0.2$ eV and 14 K; **e** The autocorrelation results calculated from **d**; **f** The intensity distribution curves along cut 1, cut 2 and cut 3 in **e**.

Supplementary References

[S1] K. McElroy, G.-H. Gweon, S. Zhou, J. Graf, S. Uchida, H. Eisaki, H. Takagi, T. Sasagawa, D.-H. Lee, and A. Lanzara, Physical review letters **96**, 067005 (2006).

[S2] M. Hashimoto, R. H. He, J. P. Testaud, W. Meevasana, R. G. Moore, D. H. Lu, Y. Yoshida, H. Eisaki, T. P. Devereaux, Z. Hussain, and Z. X. Shen, Physical Review Letters (2011).

[S3] U. Chatterjee, M. Shi, A. Kaminski, A. Kanigel, H. Fretwell, K. Terashima, T. Takahashi, S. Rosenkranz, Z. Li, H. Raffy, *et al.*, Physical review letters **96**, 107006 (2006).

[S4] R. Markiewicz, Physical Review B **69**, 214517 (2004).

[S5] Z. Jiang, H. Ma, W. Xia, Q. Xiao, Z. Liu, Z. Liu, Y. Yang, J. Ding, Z. Huang, J. Liu, *et al.*, arXiv preprint arXiv:2208.01499 (2022).

[S6] H. Li, H. Zhao, B. R. Ortiz, T. Park, M. Ye, L. Balents, Z. Wang, S. D. Wilson, and I. Zeljkovic, Nature Physics **18**, 265 (2022).

[S7] F. Tang, H. C. Po, A. Vishwanath, and X. Wan, Nature **566**, 486 (2019).

[S8] H. C. Po, Journal of Physics: Condensed Matter **32**, 263001 (2020).
